# Personalized Interpretation and Clinical Translation of Genetic Variants Associated With Cardiomyopathies

**DOI:** 10.3389/fgene.2019.00450

**Published:** 2019-05-15

**Authors:** Oscar Campuzano, Anna Fernandez-Falgueras, Georgia Sarquella-Brugada, Sergi Cesar, Elena Arbelo, Ana García-Álvarez, Paloma Jordà, Monica Coll, Victoria Fiol, Anna Iglesias, Alexandra Perez-Serra, Jesus Mates, Bernat del Olmo, Carles Ferrer, Mireia Alcalde, Marta Puigmulé, Irene Mademont-Soler, Ferran Pico, Laura Lopez, Coloma Tiron, Josep Brugada, Ramon Brugada

**Affiliations:** ^1^Cardiovascular Genetics Center, Biomedical Research Institute of Girona, University of Girona, Girona, Spain; ^2^Department of Medical Science, School of Medicine, University of Girona, Girona, Spain; ^3^Centro Investigación Biomédica Red Enfermedades Cardiovasculares, Madrid, Spain; ^4^Department of Biochemistry and Molecular Genetics, Hospital Clinic, Barcelona, Spain; ^5^Arrhythmias Unit, Hospital Sant Joan de Déu, University of Barcelona, Barcelona, Spain; ^6^Arrhythmias Unit, Hospital Clinic, University of Barcelona, Barcelona, Spain; ^7^Cardiology Service, Hospital Josep Trueta, University of Girona, Girona, Spain

**Keywords:** sudden cardiac death, arrhythmias, cardiomyopathies, genetics, genetic counseling

## Abstract

Cardiomyopathies are a heterogeneous group of inherited cardiac diseases characterized by progressive myocardium abnormalities associated with mechanical and/or electrical dysfunction. Massive genetic sequencing technologies allow a comprehensive genetic analysis to unravel the cause of disease. However, most identified genetic variants remain of unknown clinical significance due to incomplete penetrance and variable expressivity. Therefore, genetic interpretation of variants and translation into clinical practice remain a current challenge. We performed retrospective comprehensive clinical assessment and genetic analysis in six families, four diagnosed with arrhythmogenic cardiomyopathy, and two diagnosed with hypertrophic cardiomyopathy (HCM). Genetic testing identified three rare variants (two non-sense and one small indel inducing a frameshift), each present in two families. Although each variant is currently classified as pathogenic and the cause of the diagnosed cardiomyopathy, the onset and/or clinical course differed in each patient. New genetic technology allows comprehensive yet cost-effective genetic analysis, although genetic interpretation, and clinical translation of identified variants should be carefully done in each family in a personalized manner.

## Introduction

Sudden cardiac death (SCD) is a major cause of death with an incidence ranging from approximately 1:1000 to 1:3000 individuals per year in the general population (Deo and Albert, 2012). In population <35 years old, inherited cardiomyopathies are the leading cause of SCD, mainly hypertrophic cardiomyopathy (HCM), dilated cardiomyopathy (DCM), and arrhythmogenic cardiomyopathy (ACM) (Straus et al., 2004). Cardiomyopathies are characterized by structural heart abnormalities that induce mechanical and/or electrical dysfunction, leading to ventricular arrhythmias and even SCD. Sometimes the first manifestation of disease is SCD, which usually occurs in a healthy young individual while doing exercise. These disorders induce progressive morphological and histological myocardial features, but abnormalities may be minimal or even absent at young ages (Gopinathannair et al., 2015).

In post-mortem cases, current guidelines recommend molecular autopsy in all cases without a conclusive cause of death and without any cardiac alteration, but also if a cardiac alteration suspicious of cardiomyopathy is identified (Priori and Blomstrom-Lundqvist, 2015). In addition, ongoing clinical surveillance for family members of affected individuals is recommended because some relatives might have inherited the genetic alteration and thus be at risk of SCD. Therefore, establishing a proper pedigree including both clinical and genetic data is crucial to provide a clear view of diagnosed and asymptomatic relatives, focused on adopting preventive therapeutic measures in each individual. Hence, incorporation of genetic testing and genetic counseling is now considered standard of care for cardiomyopathies (Marian and Braunwald, 2017).

Massive sequencing technologies such as next generation sequencing (NGS) now allow comprehensive genetic analysis of families in a cost-effective way – genetic data can be obtained in reduced time and at low cost in comparison to traditional Sanger sequencing (Morini et al., 2015). The main current challenge is genetic interpretation as well as clinical translation of these results, as most identified genetic variants remain of unknown/ambiguous significance (Richards et al., 2015; Amendola et al., 2016). This means genetic data are not conclusive and do not help clinicians in the diagnosis, risk stratification, or adoption of potential therapeutic measures to prevent malignant arrhythmias. Consequently, genetic data should be carefully translated into clinical practice.

Information of family history, segregation of genetic variants, and genotype-phenotype correlations are crucial to elucidate the role of each genetic variant (Campuzano et al., 2015a). Further, the same genetic variant may be associated with incomplete penetrance and variable expressivity, intrafamilial as well as interfamilial, and complicating clinical interpretation (Giudicessi and Ackerman, 2013).

## Materials and Methods

This study was approved by the Ethics Committee of the Hospital Josep Trueta (Girona, Spain), following the Helsinki II declaration. Written informed consent was obtained from all relatives included in the study. Alive individuals were clinically evaluated at Hospital Josep Trueta (Girona, Spain), Hospital Clinic of Barcelona (Barcelona, Spain), or Hospital Sant Joan de Deu (Barcelona, Spain). All patients were Caucasian and native from Spain.

We analyzed six families diagnosed with cardiomyopathies caused by 3 variants in 6 families. Each variant in two families. A multidisciplinary group including cardiologists, pediatricians, genetic counselors, molecular geneticists, and pathologists performed personalized genetic interpretation and clinical translation in each family. Families diagnosed with the same cardiomyopathy due to the same mutation were included in our study. Clinical evaluation of index cases and all available relatives included a complete physical examination, 12-lead electrocardiogram (ECG), 2-dimentional echocardiography (ECHO), cardiac magnetic resonance imaging (MRI), exercise stress test, 24-h Holter monitoring, and comprehensive genetic testing. Regarding post-mortem samples, a complete autopsy was performed according to current international regulations, including macroscopic, microscopic, and toxicological analyses (Basso et al., 2008; Sinard, 2013). When macroscopic autopsy was labeled negative, forensic pathologists performed a complete histological and toxicological investigation, and collected a blood sample for genetic investigation. We excluded cases in which the autopsy was labeled as violent death, including death from drug overdose. We also obtained family history of syncope and unexplained deaths.

### Variant Analysis

Genomic DNA was obtained from peripheral blood or saliva. DNA analyzed using NGS technology (MiSeq, Illumina). We screened the most prevalent genes involved in cardiomyopathies and other pathologies associated with SCD (*ABCC9, ACTC1, ACTN2, AKAP9, ANK2, ANKRD1, BAG3, CACNA1C, CACNA2D1, CACNA1G, CACNA1H, CACNA1I, CACNB2, CALM1, CALM2, CALM3, CALR3, CASQ2, CAV3, CRYAB, CSRP3, CTNNA3, GJA1, CTF1, DES, DMD, DMPK, DPP6, DSC2, DSG2, DSP, DTNA, ECE1, EMD, EN1, EYA4, FHL2, FKTN, FLNA, FLNC, GAA, GJA5, GLA, GPD1L, HCN1, HCN2, HCN4, JPH2, JUP, KCNA5, KCND3, KCNE1, KCNE2, KCNE3, KCNE4, KCNE5, KCNH2, KCNJ2, KCNJ5, KCNJ8, KCNQ1, LAMA4, LAMP2, LDB3, LMNA, MYBPC3, MYH6, MYH7, MYL2, MYL3, MYLK2, MYOZ2, MYPN, NEBL, NEXN, NOS1AP, NOTCH1, NPPA, NUP155, PDLIM3, PHOX2A, PHOX2B, PITX2, PKP2, PLN, PRKAG2, RANGRF, RBM20, RYR2, SCN1B, SCN2B, SCN3B, SCN4B, SCN5A, SCN10A, SDHA, SGCD, SLC22A5, SLC6A4, SLC8A1, SLMAP, SLN, SNTA1, TAZ, TCAP, TGFB3, TLX3, TMEM43, TMPO, TNNC1, TNNI3, TNNT2, TP63, TPM1, TRDN, TRIM63, TRPM4, TTN, TTR*, and *VCL)* (Campuzano et al., 2014a). All gene isoforms described in Ensembl 75^1^ that have been linked at least with either a RefSeq code^2^ or CCDS^3^ were included.

Coordinates of sequence data were based on UCSC human genome version hg19 (NCBI GRCh37 built). Biotinylated cRNA probe solution was used as a capture probe (Agilent Technologies, Santa Clara, CA, United States). Bioinformatic processing of fastq files consisted of low-quality base-trimming followed by a mapping step with BWA-MEM. After removal of duplicates and low-quality reads, variant call was performed with SAMtools v.1.2, and an *ad hoc* pipeline to generate raw VCF files. The final annotation steps provided information included in public databases. Identified variations were compared with DNA sequences from 500 healthy Spanish individuals (individuals not related to any index case and of the same ethnicity) as control cases in order to identify potential founder mutations. In addition, in order to determinate the frequency of variants identified, they were contrasted with the Human Gene Mutation Database (HGMD^4^), HapMap^5^, 1000 Genomes Project^6^, Exome Variant Server (EVS^7^), Genome Aggregation Database (gnomAD^8^), and Exome Aggregation Consortium (ExAC^9^). Non-common genetic variants [minor allele frequency (MAF) <1%] identified in NGS analysis were confirmed by Sanger sequencing. Exons and exon-intron boundaries of each gene were amplified (Verities PCR, Applied Biosystems, Austin, TX, United States). PCR products were purified (Exosap-IT, Affymetrix Inc., USB Products, Cleveland, OH, United States), and then were directly sequenced in both directions (Big Dye Terminator v3.1 and 3130XL Genetic Analyzer, Applied Biosystems) with posterior SeqScape Software v2.5 analysis (Life Technologies, Carlsbad, CA, United States), comparing obtained results with the hg19 reference sequence. Sequence variants were described following rules outlined by the Human Genome Variation Society (HGVS^10^), and checked in Mutalyzer^11^. *In silico* prediction of pathogenicity of genetic variations was assessed in Functional Annotations for Non Synonymous SNVs (FannsDB^12^), Mutation Taster^13^, Protein Variation Effect Analyzer (PROVEAN^14^), and Polymorphism Phenotyping v2 (PPH2^15^). Alignment of DNA sequences for different species was also performed for novel variations using Uniprot database^16^. Finally, protein structure and domains were consulted at STRING^17^ and SMART databases^18^.

Regarding copy number variation (CNV), our approach focused on capturing significant differences between expected normalized coverage and obtained normalized coverage for a given sample in a region of interest. Several samples were analyzed to corroborate similar levels of coverage between samples, as already published by our group (Campuzano et al., 2014b). All CNVs were compared with ExAC, including recently added data concerning CNVs. Genetic analysis in relatives was performed using the Sanger method. Only rare genetic variants confirmed in the index case were analyzed in relatives to perform a comprehensive genetic study in each family.

Finally, each variant was classified in one of the following current recommendations of the American College of Medical Genetics and Genomics and the Association for Molecular Pathology (ACMG/AMP) (Richards et al., 2015). This recommendations describe several items of pathogenicity (PVS, evidence of pathogenicity very strong; PS, evidence of pathogenicity strong; PM, evidence of pathogenicity moderate; and PP, evidence of pathogenicity supporting), and benignity (BA, evidence of benign impact stand-alone; BS, evidence of benign impact strong; and BP, evidence of benign impact supporting), enabling a final score and consequent classification of variants as: pathogenic (P), likely pathogenic (LP), variant of uncertain significance (VUS), likely benign (LB), or benign (B). The PM2 item in the ACMG score was considered fulfilled if MAF ≤0.1% in relevant population databases (Lek et al., 2016). Concerning frequency of disease-causing variants, the majority of pathogenic variants are extremely rare (<0.01%) (Kobayashi et al., 2017). Concerning potential PVS1 variants, it should be only used for variants in genes where loss of function is a previously established disease mechanism^19^ (Abou Tayoun et al., 2018). In addition, some items of ACMG/AMP may underlie a lack of specificity or ambiguous or contradictory interpretations, so we check the parameters using *Sherloc* (semiquantitative, hierarchical evidence-based rules for locus interpretation) (Nykamp et al., 2017). Finally, to avoid bias, three authors with PhDs in human genetics independently and comprehensively investigated published genetic data concerning each analyzed variant. In addition, four independent expert cardiologists (including two pediatric cardiologists) comprehensively reviewed available clinical data to reconfirm diagnoses following current guidelines (Priori and Blomstrom-Lundqvist, 2015). All investigators discussed data included in each item of the ACMG and consensus and the final classification of all variants.

## Results

**FIGURE 1:**
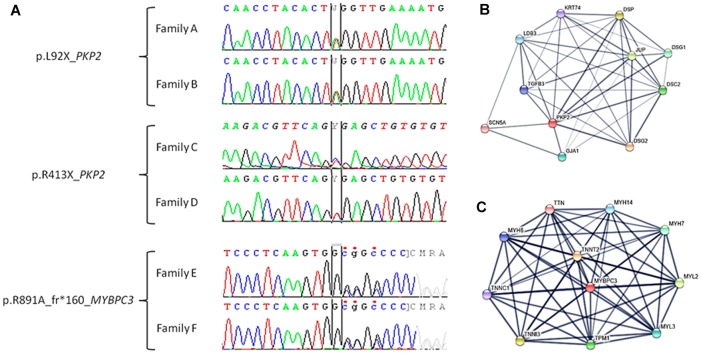
Electropherogram of pathogenic variants identified in our families and closest proteins interactions. **(A)** Families A and B: c.275T > A (TTG > TAG), p.L92X (p.Leu92^∗^) in the *PKP2* gene. Families C and D: c.1237C > T (CGA > TGA) and p.R413X (p.Arg413^∗^) in the *PKP2* gene. Families E and F: c.2670dupG, p.R891A_frX160 (p.Arg891Ala_fs^∗^160) in the *MYBPC3* gene. **(B)** Network of ten closest proteins to PKP2. **(C)** Network of ten closest proteins to MYBPC3.

Our study retrospectively analyzed six families diagnosed with cardiomyopathy after comprehensive clinical assessment – families A, B, C, and D included relatives clinically diagnosed with ACM, and families E and F included relatives diagnosed with HCM. Comprehensive NGS analysis was performed on at least one patient clinically diagnosed with ACM or HCM in each family. Concerning NGS data, all samples showed <4% duplicates of amplified regions; a minimum of 96% reads mapped uniquely and 55% reads of insert. Finally, the median coverage was 300× and call rate at 30× was 99.9%. After genetic interpretation, three pathogenic rare variants – each present in two different families – were identified as the cause of disease. Families A and B carried the variant *PKP2*_p.(Leu92^∗^), families C and D carried the variant *PKP2*_p.(Arg413^∗^), and families E and F carried the variant *MYBPC3*_p.(Arg891Ala_fr^∗^160). Variants *PKP2*_p.(Leu92^∗^) and *PKP2*_p.(Arg413^∗^) are previously reported *non-sense* variants that are widely accepted as pathogenic. Both genetic abnormalities radically change the protein, resulting in a final protein that is shorter than wildtype PKP2 (Figure 1 and Tables 1, 2).

**Table 1:** Clinical data.

Family	Individual	Age	Gender	Gene_Variant	Diagnosis	Criteria
A	II.1	74	F	*PKP2_*p.(Leu92^∗^)	No	–
	III.1	48	F	*PKP2_*p.(Leu92^∗^)	ACM	Definite (2 major and 2 minor)
	III.4	42	F	*PKP2_*p.(Leu92^∗^)	ACM	Definite (1 major and 2 minor)
B	II.1	68	F	*PKP2_*p.(Leu92^∗^)	ACM	(1 major and 1 minor)
	III.1	39	F	*PKP2_*p.(Leu92^∗^)	ACM	Definite (1 major and 2 minor)
C	I.1†	44	M	?	ACM	Possible (2 minor)
	II.2	42	F	*PKP2_*p.(Arg413^∗^)	ACM	Definite (2 major and 2 minor)
	II.3†	21	M	*PKP2_*p.(Arg413^∗^)	ACM	Definite (1 major and 2 minor)
	II.4	40	M	*PKP2_*p.(Arg413^∗^)	ACM	Definite (2 major and 1 minor)
	II.6	37	M	*PKP2_*p.(Arg413^∗^)	ACM	Definite (2 major and 2 minor)
	III.2	9	M	*PKP2_*p.(Arg413^∗^)	ACM	Definite (1 major and 2 minor)
D	II.1	74	F	*PKP2_*p.(Arg413^∗^)	ACM	Definite (2 major and 1 minor)
	II.5	71	M	*PKP2_*p.(Arg413^∗^)	ACM	Definite (2 major and 1 minor)
	II.7	68	M	*PKP2_*p.(Arg413^∗^)	ACM	Definite (1 major and 2 minor)
	III.2	39	F	*PKP2_*p.(Arg413^∗^)	ACM	Definite (1 major and 2 minor)
	III.4	35	F	*PKP2_*p.(Arg413^∗^)	No	–
	III.6	30	M	*PKP2_*p.(Arg413^∗^)	ACM	Definite (2 major and 1 minor)
	III.8	38	M	*PKP2_*p.(Arg413^∗^)	ACM	Definite (2 major and 1 minor)
	IV.1†	10	M	*PKP2_*p.(Arg413^∗^)	ACM	(2 minor)
	IV.2	11	M	*PKP2_*p.(Arg413^∗^)	No	–
	IV.5	6	F	*PKP2_*p.(Arg413^∗^)	No	–
E	II.2	68	F	*MYBPC3*_p.(Arg891Alafs^∗^160)	HCM	LV wall thickness 19 mm
	II.3	66	M	*MYBPC3*_p.(Arg891Alafs^∗^160)	HCM	LV wall thickness 20 mm
	III.2	39	M	*MYBPC3*_p.(Arg891Alafs^∗^160)	HCM	LV wall thickness 17 mm
	III.4	34	F	*MYBPC3*_p.(Arg891Alafs^∗^160)	No	–
	III.6†	38	M	*MYBPC3*_p.(Arg891Alafs^∗^160)	HCM	Myocardial disarray
	III.8	36	M	*MYBPC3*_p.(Arg891Alafs^∗^160)	HCM	LV wall thickness 24 mm
	III.11	30	M	*MYBPC3*_p.(Arg891Alafs^∗^160)	HCM	LV wall thickness 16 mm
	IV.2	11	M	*MYBPC3*_p.(Arg891Alafs^∗^160)	No	–
	IV.4	8	F	*MYBPC3*_p.(Arg891Alafs^∗^160)	No	–
	IV.6	3	F	*MYBPC3*_p.(Arg891Alafs^∗^160)	No	–
	IV.8	2	F	*MYBPC3*_p.(Arg891Alafs^∗^160)	No	–
F	II.2	73	M	*MYBPC3*_p.(Arg891Alafs^∗^160)	HCM	LV wall thickness 25mm
	III.1	46	F	*MYBPC3*_p.(Arg891Alafs^∗^160)	HCM	LV wall thickness 23mm
	III.2	41	M	*MYBPC3*_p.(Arg891Alafs^∗^160)	HCM	LV wall thickness 22mm


*ACM, arrhythmogenic cardiomyopathy; F, female; HCM, hypertrophic cardiomyopathy; LV, left ventricle; M, male. Sign † means sudden death.*

**Table 2:** Genetic data of variations.

Family	Gene	Nucleotide	Protein	dbSNP	EVS	ExAC	gnomAD	PP2	PROVEAN	MuTa	FansDB	HGMD (disease)	ClinVar (pathology)	ACMG score
A	*PKP2*	c.275T > A	p.(Leu92^∗^)	rs763639737	NA	NA	2/251424	NA	NA	NA	D	CM102825 (ACM)	P (ACM)	P
	*DSG2*	c.527C > T	p.(Thr176Ile)	rs536617217	NA	2/120200	2/28069	B	N	DC	N	NA	VUS	VUS
B	*PKP2*	c.275T > A	p.(Leu92^∗^)	rs763639737	NA	NA	2/251424	NA	NA	NA	D	CM102825 (ACM)	P (ACM)	P
C	*PKP2*	c.1237C > T	p.(Arg413^∗^)	rs372827156	1/13005	2/121408	4/282766	NA	NA	NA	D	CM060431 (ACM)	P (ACM)	P
D	*PKP2*	c.1237C > T	p.(Arg413^∗^)	rs372827156	1/13005	2/121408	4/282766	NA	NA	NA	D	CM060431 (ACM)	P (ACM)	P
	*MYH7*	c.4772T > A	p.(Leu1591Gln)	rs730880808	NA	7/121931	10/251476	B	N	DC	N	CM1413450 (SCD)	VUS	VUS
	*TTN*	c.60754G > C	p.(Ala20252Pro)	rs72646880	27/12015	236/120342	513/279434	PD	D	DC	N	NA	VUS	LB
E	*MYBPC3*	c.2670dupG	p.(Arg891Alafs^∗^160)	Novel	NA	NA	NA	NA	NA	DC	D	NA	NA	P
F	*MYBPC3*	c.2670dupG	p.(Arg891Alafs^∗^160)	Novel	NA	NA	NA	NA	NA	DC	D	NA	NA	P


*ACM, arrhythmogenic cardiomyopathy; ACMG, American College of Medical Genetics and Genomics; B, benign; D, deleterious; DC, disease causing; EVS, exome variant server; ExAC, exome aggregation consortium; HGMD, human gene mutation database; MuTa, mutation taster; NA, not available; N, neutral; P, pathogenic; PD, possibly damaging; PP2, PolyPhen2; SCD, sudden cardiac death; VUS, variant uncertain/unknown significance.*

The *PKP2*_p.(Leu92^∗^) variant was reported first as a cause of ACM in a cohort of cases (Fressart et al., 2010). Currently, global population databases have reported this variant with very low frequency (2/251424, MAF: 0.0007955%), and *in silico* analysis predicts this genetic alteration is deleterious. The *PKP2*_p.(Arg413^∗^) variant was reported for the first time in 2006 in a cohort of cases with ACM (Syrris et al., 2006). To date, more than 10 reports describe families with ACM due to this pathogenic variant, and *in silico*, analysis predicts the variant is deleterious. Despite identification of this variant in global databases (rs372827156), the frequency is very low (2/121408 alleles, MAF: 0.001647%). The third variant, *MYBPC3*_p.(Arg891Ala_fr^∗^160), is novel. Current global population databases do not include this variant, and *in silico* analyses predict a deleterious role. It is a small *indel* that induces a frameshift, resulting in a longer than wildtype protein. Therefore, all parameters analyzed indicate a pathogenic role (Figure 1 and Tables 1, 2).

### Families A and B

Families A and B were clinically diagnosed with ACM (Table 1). The cause of the disease was a pathogenic variant (CM102825) in *PKP2* (c.275T > A, p.L92X – p.Leu92^∗^) (Table 2). Our NGS panel included genes encoding the functionally closest PKP2-related proteins, but no abnormalities were identified except in Family A. In addition, no CNVs were identified in either family.

**FIGURE 2:**
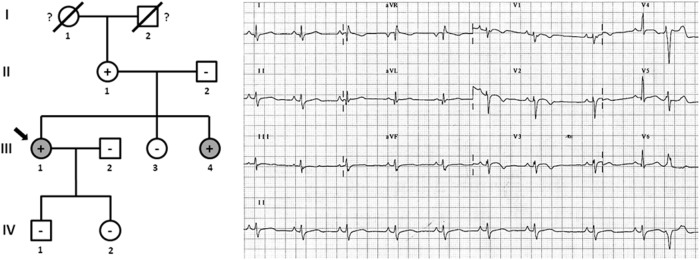
Pedigree of family (A). Generations are indicated in the left side. Each individual is identified with a number. Clinically affected patients are show in gray, clinically unaffected patients are show in white, and slashes indicate a deceased relative. Index case is indicated with an arrow (III.1). The genetic carriers of *PKP2_*p.(Leu92^∗^) are represented with a plus sign inside rounds/squares. Minus sign indicates non-carriers of the genetic variant. Question mark indicates no genetic analysis available. The ECG corresponds to index case. The ECG showing negative T waves in V1-3, and prolonged Terminal Activation Duration as major and respectively, minor diagnostic Task Force Criteria for ACM.

Family A included two members clinically diagnosed with ACM, III.1 -48 years old- and III.4 -42 years old- (Figure 2). Individual III.1 had repeated episodes of palpitations. During clinical assessment (right ventricle ejection fraction: 38%; dilated right ventricle: 4.3 cm; no left ventricle affectation), III.1 suffered a syncope due to ventricular arrhythmia, and an implantable cardioverter defibrillator (ICD) was implanted. Individual III.4 was diagnosed with ACM -abundant fibro-fatty infiltration in epicardium and myocardium of right ventricle (residual myocytes <60%), moderate decrease of right ventricular ejection fraction (45%), and mild right ventricle dilatation-, but with a slow clinical evolution during follow-up and negligible symptoms to date. No other relatives were diagnosed with ACM except one family member (II.1 -74 years old-) who showed scattered cardiac fibrosis but not a definite diagnosis of ACM. She remained asymptomatic thus far, without any episode of palpitations, arrhythmia, or syncope. Neither SCD nor syncope episodes occurred in this family. The grandparents (I.1 and I.2) died at old age (>75 years old both), both due to oncologic diseases, and without documented episodes of syncope. Genetic analysis identified the already reported pathogenic variant -*PKP2*_p.(Leu92^∗^)- in three cases, II.1, III.1, and III.3. In addition, a rare variant in *DSG2* (c.527C > T, p.T176I – p.Thr176Ile) was also identified in the index case (III.1), with the amino acid threonine (polar, uncharged side chain) changed to isoleucine (non-polar, hydrophobic side chain). This variant is reported in global databases with a low frequency (2/249296, MAF: 0.0008023%). However, *in silico* analysis predicts an ambiguous role, and alignment between species showed no highly conserved domain. The variant in *DSG2* was present in the index case (III.1, clinically diagnosed with ACM), her sister (III.3 -44 years old-, not diagnosed with ACM), and her mother (II.1, not diagnosed with ACM but showing scattered cardiac abnormalities). One relative clinically diagnosed with ACM (III.4) did not carry this variant. Therefore, we disregarded *DSG2*_p.(Thr176Ile) as pathogenic, at least as the cause of ACM in this family.

**FIGURE 3:**
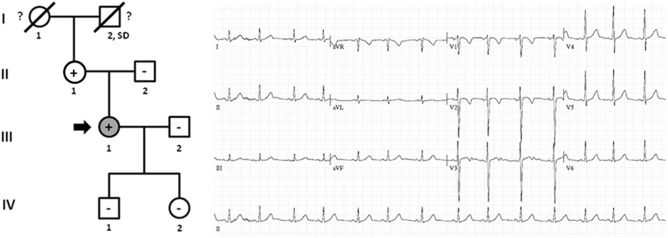
Pedigree of family B. Generations are indicated in the left side. Each individual is identified with a number. Clinically affected patients are shown in gray, clinically unaffected patients are shown in white, and slashes indicate a deceased relative. Index case is indicated with an arrow (III.1). The genetic carriers of *PKP2_*p.(Leu92^∗^) are represented with a plus sign inside rounds/squares. Minus sign indicates non-carriers of the genetic variant. Question mark indicates no genetic analysis available. SD means Sudden Death. The ECG corresponds to index case. ECG showing negative T waves in V1-2 (minor ACM criterion).

Family B also carried the same *non-sense* variant, *PKP2*_p.(Leu92^∗^). Relative III.1-39 years old- was definitely diagnosed with ACM (Figure 3). She suffered syncope during exercise. Clinical assessment showed dyskinesia with a moderate dilation of the right ventricle (39 mm), a minor reduction of ejection fraction (40%), and no criteria of fibro-adipose infiltration in the myocardium (residual myocytes nearly 75% in right ventricle). The left ventricle was not affected. Given the risk of lethal arrhythmias, this individual had an ICD implanted 6 years ago, with three appropriate shocks registered to date. Individual II.1 -68 years old-, despite old age, showed no symptoms during most of her life (punctual palpitations in the past 8 years during moderate physical activity). One relative (grandfather, I.2) died suddenly at a young age -36 years old- while working on a farm. However, neither complete autopsy nor molecular autopsy was performed. No other rare variants were identified in this family.

### Families C and D

Families C and D were also diagnosed with ACM (Table 1), and both carried a previous reported pathogenic variant (CM060431) in *PKP2* (c.1237C > T, p.R413X – p.Arg413^∗^), associated with ACM (Table 2). Our NGS panel included genes encoding the closest PKP2-related proteins, but no abnormalities were identified in these genes. In addition, no CNVs were identified in either family.

**FIGURE 4:**
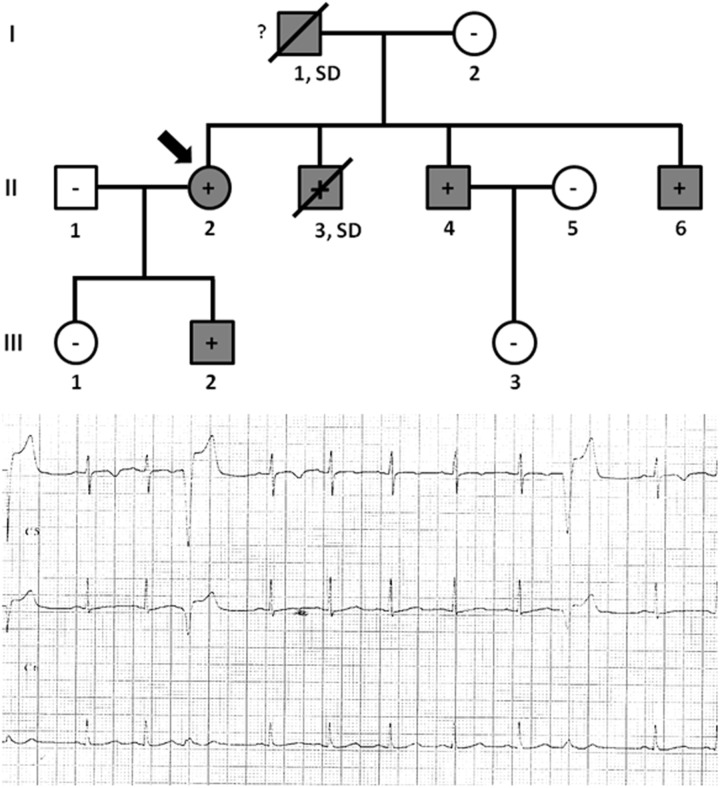
Pedigree of family C. Generations are indicated in the left side. Each individual is identified with a number. Clinically affected patients are shown in gray, clinically unaffected patients are shown in white, and slashes indicate a deceased relative. Index case is indicated with an arrow (II.2). The genetic carriers of *PKP2_*p.(Arg413^∗^) are represented with a plus sign inside rounds/squares. Minus sign indicates non-carriers of the genetic variant. Question mark indicates no genetic analysis available. SD means Sudden Death. The ECG corresponds to index case.

Family C included six family members diagnosed with ACM, four of which were alive (II.2 -42 years old-, II.4 -40 years old-, II.6 -37 years old-, and III.2 -9 years old-) and carried the same genetic variant, *PKP2*_p.(Arg413^∗^) (Figure 4). Concerning diagnosis, inverted T-wave in right precordial leads were observed in three cases (II.2, II.4, and II.6); in addition, sustained ventricular tachycardia were observed in II.4, and II.6. Image analysis in II.2 showed regional RV akinesia and 38% RV ejection fraction. In III.2, regional RV akinesia and PLAX RV outflow tract of 31 mm were identified. The other two relatives died suddenly at a young age and both were diagnosed with ACM post-mortem (both showing residual myocytes <60% in fibro-fatty infiltration); I.1 died working in a building site -44 years old-, and II.3 died at young age during exercise -21 years old-. Molecular autopsy indicated individual II.3 carried the same genetic variant. Unfortunately, no sample was obtained from I.1 to perform genetic analysis. No other rare variants were identified in this family.

**FIGURE 5:**
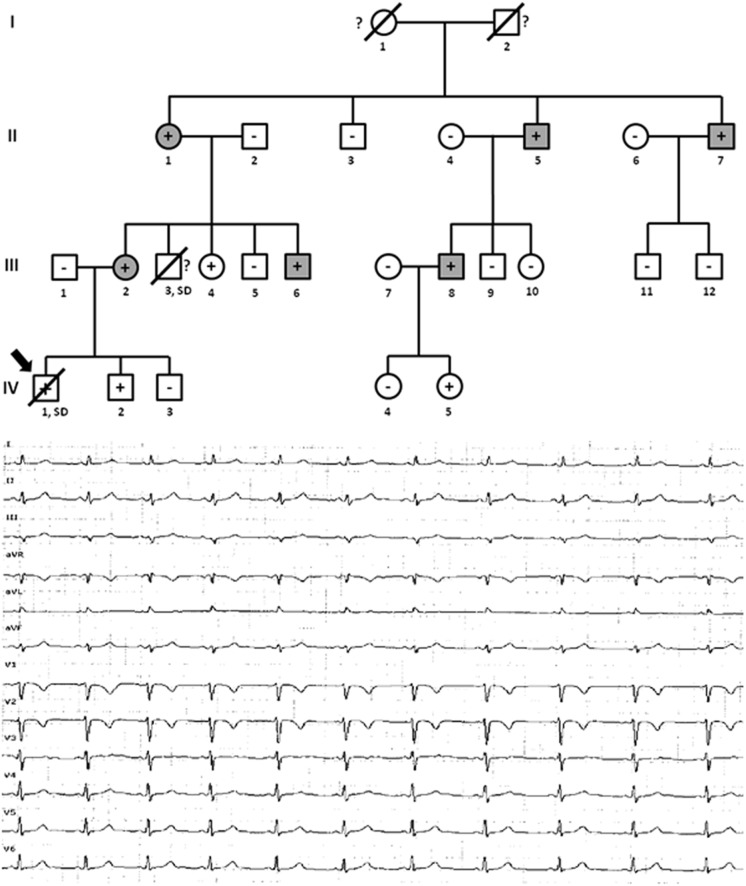
Pedigree of family D. Generations are indicated in the left side. Each individual is identified with a number. Clinically affected patients are shown in gray, clinically unaffected patients are shown in white, and slashes indicate a deceased relative. Index case is indicated with an arrow (IV.1). The genetic carriers of *PKP2_*p.(Arg413^∗^) are represented with a plus sign inside rounds/squares. Minus sign indicates non-carriers of the genetic variant. Question mark indicates no genetic analysis available. SD means Sudden Death. The ECG corresponds to IV.1.

Family D included six alive family members diagnosed with ACM (II.1 -74 years old-, II.5 -71 years old-, II.7 -68 years old-, III.2 -39 years old-, III.6 -30 years old-, and III.8 -38 years old-) (Figure 5). Inverted T-wave in right precordial leads were observed in five relatives (II.1, II.5, III.2, III.6, and III.8). III.7 also showed inverted T-wave in right precordial leads but in the presence of complete right bundle-branch block. Non-sustained ventricular tachycardia of left bundle-branch morphology were observed in III.6, and III.8. Image analysis showed RV ejection fraction of 38% and 39% in II.1 and II.5, respectively. All other relatives (II.7, II.2, III.6, and III.8) showed RV ejection fraction between 40 and 45%. Two relatives (III.3 and IV.1) died at a young age (15 and 10 years old, respectively). The index case, IV.1, died while sleeping and showed minor post-mortem cardiac abnormalities (including myocarditis) with no definite diagnosis of ACM but possible. Neither autopsy nor molecular autopsy was performed in III.3 despite SCD at a young age during exercise. No syncope or cardiac pathology was diagnosed in either young relative who died suddenly. Molecular autopsy of the index case (IV.1) identified three rare genetic variants –*PKP2*_p.(Arg413^∗^), *MYH7*_p.(Leu1591Gln) and *TTN*_p.(Ala20252Pro)-. All clinically affected relatives (II.1, II.5, II.7, III.2, III.6, and III.8) carried the *PKP2*_p.(Arg413^∗^) variant. Three additional relatives (III.4 -35 years old-, IV.2 -11 years old-, and IV.5 -6 years old-) carried the same genetic variant, but all remain asymptomatic so far. Individual III.4 showed some cardiac abnormalities, although they were not diagnostic of ACM following current guidelines (Marcus et al., 2010). Neither grandparent (I.1 nor I.2) died suddenly or at a young age (both more than 75 years old). Concerning the other two rare variants identified -*MYH7*_p.(Leu1591Gln) and *TTN*_p.(Ala20252Pro)-, previous studies published by our group disregard a pathogenic role (Campuzano et al., 2015b).

### Families E and F

Families E and F were diagnosed with HCM (Table 1). The disease was caused by a novel variant in *MYBPC3*. This variant is a duplication of G (c.2670dupG), inducing a frameshift (p.Arg891Ala_fr^∗^160) (Table 2). This genetic alteration induces a radical change in fibronectin type 3 (FN3) domains, changing the amino acid sequence of wildtype MYBPC3 starting at amino acid 891. Our NGS panel included genes encoding the closest MYBPC3-related proteins, but no abnormalities were identified. In addition, neither other rare variants nor CNVs were identified in either family.

**FIGURE 6:**
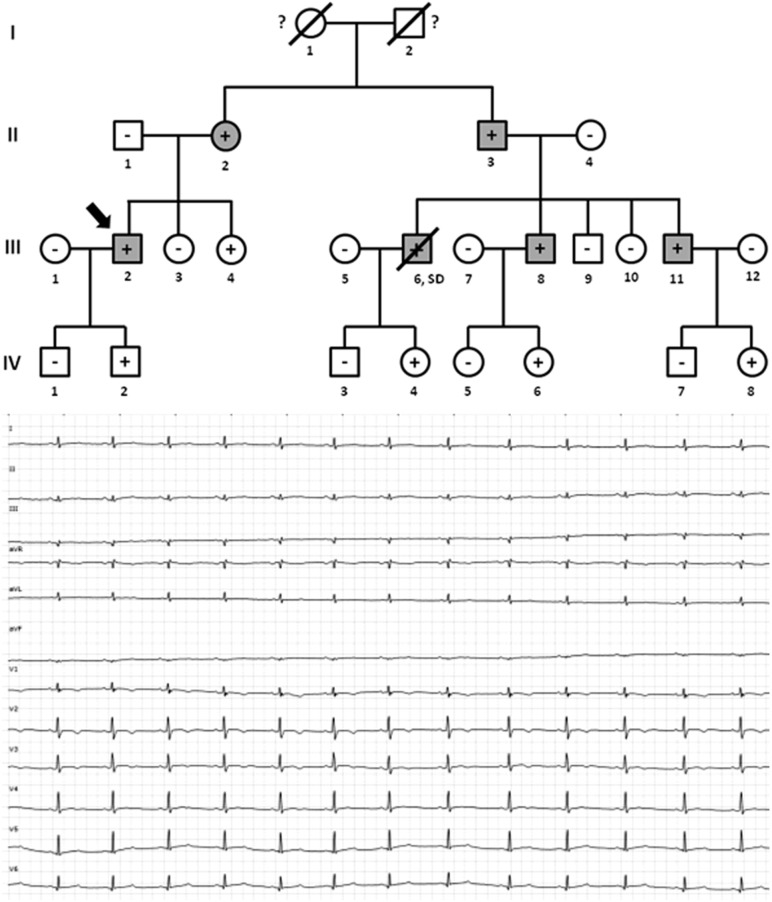
Pedigree of family E. Generations are indicated in the left side. Each individual is identified with a number. Clinically affected patients are shown in gray, clinically unaffected patients are shown in white, and slashes indicate a deceased relative. Index case is indicated with an arrow (III.2). The genetic carriers of *MYBPC3*_p.(Arg891Alafs^∗^160) are represented with a plus sign inside rounds/squares. Minus sign indicates non-carriers of the genetic variant. Question mark indicates no genetic analysis available. SD means Sudden Death. The ECG corresponds to index case.

Family E included five living family members (II.2 -68 years old-, II.3 -66 years old-, III.2 -39 years old-, III.8 -36 years old-, and III.11 -30 years old-) diagnosed with HCM (Figure 6). Only III.8 (left ventricular wall thickness 24 mm; ejection fraction 41%), had syncope and an ICD was implanted. All other relatives were diagnosed with HCM: II.2 -left ventricular wall thickness 19 mm; ejection fraction 49%-, II.3 -left ventricular wall thickness 20 mm; ejection fraction 45%-, III.2 -left ventricular wall thickness 17 mm; ejection fraction 49%-, and III.11 -left ventricular wall thickness 16 mm; ejection fraction 51%-. They did not show severe heart abnormalities (follow-up showed slow evolution of disease), and no syncope has been documented thus far. All carried the same rare genetic variant, *MYBPC3*_p.(Arg891Ala_fr^∗^160). Individual III.6 was diagnosed post-mortem –died at 38 years old-, during an extreme effort at work. Molecular autopsy identified the same variant (p.Arg891Ala_fr^∗^160) and forensic report described an asymmetric septal hypertrophy and microscopic analysis identified myocardial disarray with interstitial fibrosis. In addition, five relatives (III.4 -34 years old-, IV.2 -11 ears old-, IV.4 -8 years old-, IV.6 -3 years old-, and IV.8 -2 years old-) carried the same genetic variant, but all remained asymptomatic and without any cardiac alteration, so far. Both grandparents (I.1 and I.2) died at old age (both >75 years old) due to oncologic and ischemic episodes, respectively. Neither had documented episodes of arrhythmia or syncope.

**FIGURE 7:**
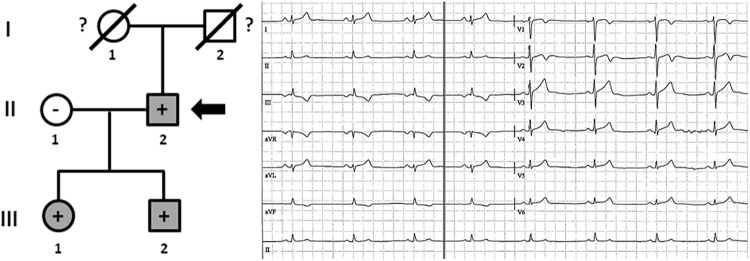
Pedigree of family F. Generations are indicated in the left side. Each individual is identified with a number. Clinically affected patients are shown in gray, clinically unaffected patients are shown in white, and slashes indicate a deceased relative. Index case is indicated with an arrow (II.2). The genetic carriers of *MYBPC3*_p.(Arg891Alafs^∗^160) are represented with a plus sign inside rounds/squares. Minus sign indicates non-carriers of the genetic variant. Question mark indicates no genetic analysis available. The ECG corresponds to index case.

Family F included three living family members (III.1, III.2, and II.2) diagnosed with HCM (Figure 7). Individual II.2 -73 years old- had a syncopal episode several years before, and an ICD was implanted. He showed a left ventricular wall thickness of 25 mm, mitral valve regurgitation, and slightly decreased ejection fraction (48%). The other two relatives (III.1 and III.2), despite young ages (46 and 41 years old, respectively), showed enlarged left ventricular wall thickness (23 mm and 22 mm, respectively), moderately decreased ejection fraction (45 and 47%, respectively), and mitral valve regurgitation. Individual III.1 had an ICD implanted due to syncope. The three living relatives carried the same genetic variant, *MYBPC3*_p.(Arg891Ala_fr^∗^160). Neither grandparent (I.1 nor I.2) died suddenly or at a young age (both >75 years old).

## Discussion

The “five P” characteristics in current medicine – personalized, predictive, preventive, participative, and precision – are an innovative approach to the patient using available technologies to interpret individually all patient data. Recently updated guidance, first published in 2009 for the Heart Failure Society of America and ACMG, supports the use of NGS in genetic testing for cardiomyopathy to assist patient care and management of at-risk family members (Hershberger et al., 2018). In our study, we carried out an individualized interpretation of three pathogenic variants leading to cardiomyopathy in six families showing high expression variability even for the same variant and family due to different onset, evolution, and/or outcome of the disease.

Families A and B were diagnosed with ACM due to a pathogenic variant, *PKP2*_p.(Leu92^∗^) (CM102825). Our group previously published the index case of Family A (Alcalde et al., 2014), and here we have included family segregation. Hence, Family A showed incomplete penetrance and variable expressivity because one relative carried the pathogenic variant but had no diagnosis of ACM (despite showing some scattered fibrotic heart abnormalities). Intriguingly, the relative without an ACM diagnosis is the progenitor of both clinically affected patients, may be due to age-dependent penetrance age. The phenotype of both diagnosed patients differed despite their similar age—neither had early disease onset, but the evolution was faster and more severe in one patient in contrast to the other. Further, the variant in *DSG2* (p.T176I) was not present in a relative clinically diagnosed with ACM (III.4). Therefore, we disregarded *DSG2*_p.(Thr176Ile) as pathogenic, at least in Family A, remaining the variant *PKP2*_p.(Leu92^∗^) is the most plausible cause of disease in Family A.

Family B, despite only two genetic carriers showed incomplete penetrance and variable expressivity. The younger relative (III.1) showed an aggressive phenotype, with often-malignant arrhythmias, and had an ICD implanted. The older relative (II.1) showed a slow evolution and benevolent phenotype due to minimal symptoms throughout her life. Therefore, considering the clinical data in both Families A and B, *PKP2*_p.(Leu92^∗^) may be considered the cause of ACM, although other genetic abnormalities may modify onset, evolution, and outcome of the disease.

Families C and D were also diagnosed with ACM and both carried a pathogenic variant (CM060431) in *PKP2* (c.1237C > A, p.R413X - p.Arg413^∗^). Our group published on Family C in 2014 (Alcalde et al., 2014). All clinically affected relatives carried the *non-sense* variant in *PKP2*, and young relatives showed cardiac abnormalities associated with ACM, suggesting an early onset of disease due to this genetic variant. In 2015, our group also published part of Family D (Campuzano et al., 2015b), and here we have included more relatives to confirm the pathogenic role of *PKP2*_p.(Arg413^∗^) as cause of disease. Genotype-phenotype correlation suggests that *PKP2*_p.(Arg413^∗^) is the cause of disease in these families, whereas additional variants could play a role as phenotype modulators. We observed incomplete penetrance and variable expressivity, as occurs in other reported families affected by ACM (Kannankeril et al., 2006). Asymptomatic patients carrying this genetic variant were young relatives. However, it is well accepted that ACM is a progressive structural disease, so cardiac abnormalities may not yet be evident in early ages. Indeed, a middle-aged relative in Family D showed some cardiac abnormalities that were not diagnostic of ACM following current guidelines (Marcus et al., 2010). Therefore, the same *non-sense* variant induces pathology in both Families C and D, but the onset in each family is different, especially in young individuals (<40 years old). In Family C, relatives showed evident heart abnormalities at an early age, suggesting a highly aggressive role of the variant; however, in Family D, the abnormalities seemed to occur progressively, suggesting a pathogenic role of the variant but not with the same temporal onset. Therefore, despite *PKP2*_p.(Arg413^∗^) being the cause of ACM, other genetic abnormalities (although not in any of the closest related proteins) may modify the onset, evolution, and outcome of ACM.

Families E and F were diagnosed with HCM due to a novel frameshift alteration in *MYBPC3* that results in a different protein than wildtype MYBPC3. All analyses suggest a pathogenic role. Family E showed incomplete penetrance and variable expressivity, with clinically diagnosed relatives carrying the *MYBPC3*_p.(Arg891Alafs^∗^160) variant and showing different phenotypic evolution of heart abnormalities. This agrees with several reports showing different phenotypes from one family diagnosed with HCM due to a single pathogenic variant (Wang et al., 2018). In Family E, asymptomatic patients were young relatives, suggesting a delay in the onset of HCM that makes cardiac manifestations not yet evident at younger ages. This agrees with previous reports suggesting that pathogenic variants in genes associated with HCM do not induce structural abnormalities at early years (Brion et al., 2009, 2012). Family F showed complete penetrance and variable expressivity. All relatives who carried the *MYBPC3*_p.(Arg891Alafs^∗^160) variant had a clinical diagnosis of HCM, including young family members. Therefore, *MYBPC3*_p.(Arg891Alafs^∗^160) is the likely cause of HCM in both Families E and F, and other genetic abnormalities (although not in the closest related proteins) may modify onset, evolution, and outcome of the disease in each family.

Autopsy in young individuals who die suddenly may be not conclusive and may not identify cardiac abnormalities. However, potential cardiomyopathy should not be disregarded, as ultramicroscopic changes that are not evident at autopsy may nonetheless induce arrhythmia responsible for SCD (Campuzano et al., 2014a). Molecular autopsy may identify the genetic alteration in a gene associated with cardiomyopathy but without anatomic heart abnormalities. This fact is important in sudden deaths occurring in infants, children, and even young people (Sarquella-Brugada et al., 2016). A recent study states that, carriers of a pathogenic variant may be at 2.5-fold greater risk of SCD compared to non-carriers (Milano et al., 2016). Nonetheless, it should be communicated to the patient that despite being a carrier, the individual may never express the disease phenotype (Kapplinger and Ackerman, 2016). Similar to our study, a 2010 publication of a cohort of 60-year-old patients diagnosed with ACM and carrying a pathogenic radical variant indicates that only 60% of genetic carriers had experienced any symptoms, without differences between genders (van der Zwaag et al., 2010). It is well-known that cardiomyopathies may display a broad range of phenotype variability, from asymptomatic individuals to severe heart alterations; it is also important to remark that sometimes the first manifestation of disease is SCD, which usually occurs in a healthy young individual while doing exercise, main trigger of life-threatening ventricular arrhythmias. Considering all data, we believe that clinical follow-up including periodic tests of risk should be performed in all genetic carriers, despite remaining asymptomatic due to high risk of lethal arrhythmias, accordingly to recent published manuscripts (Kapplinger and Ackerman, 2016). In addition, proper genetic counseling is essential for all family members despite the high complexity of genetic heterogeneity, incomplete penetrance, and variable expressivity related to inherited cardiomyopathies (De Bortoli et al., 2017). In this prospective, further genetic studies aiming to identify modifying factors are needed to improve prevention of malignant arrhythmias and guide appropriate treatment in each patient.

In conclusion, genetic testing has been progressively incorporated into clinical diagnosis, mainly due to improved NGS technology. Although genetic interpretation may classify rare variants as pathogenic, translation into clinical practice sometimes shows both interfamilial and intrafamilial phenotypic differences. We report here families carrying the same pathogenic variant associated with cardiomyopathies but with differential onset and/or clinical progression. Therefore, additional factors may modify the disease phenotype in each individual. Nonetheless, all individuals carrying a pathogenic variant, despite being asymptomatic, are at risk of disease therefore, clinical follow-up should be performed. Clinical translation remains a main challenge for cardiovascular genetic units and should be carefully performed in a personalized manner.

### Limitations

A main limitation of this study is the lack of understanding of the reasons implicated in high expression variability even for the same variant and family. Additional analysis of the assessed genes as well as in other genes not included in our NGS custom-panel is necessary to identify additional genetic variants that could modify the phenotype. In addition, other genetic analyses focused on transcriptional/proteomic modifications are necessary to clarify the role of each genetic variant in each individual. It is important to remark the absence of data on exercise, an important contributor to phenotypic manifestation. Finally, *in vivo* and/or *in vitro* studies of each genetic variant may help clarify pathophysiological mechanisms associated with the pathology.

## Ethics Statement

This study was approved by the Ethics Committee of the Hospital Josep Trueta (Girona, Spain), following the Helsinki II declaration. Written informed consent was obtained from all relatives included in the study. Alive individuals were clinically evaluated at Hospital Josep Trueta (Girona, Spain), Hospital Clinic of Barcelona (Barcelona, Spain), or Hospital Sant Joan de Deu (Barcelona, Spain).

## Author Contributions

OC, AF-F, GS-B, JB, and RB developed the concept. SC, MC, JM, EA, AG-Á, PJ, AP-S, BdO, CF, MA, VF, AI, MP, LL, FP, IM-S, and CT acquired, pre-processed, and analyzed the data. OC, AF-F, and GS-B prepared the manuscript. OC, JB, and RB supervised the study. All authors contributed to manuscript revision, read, and approved the submitted version.

## Conflict of Interest Statement

The authors declare that the research was conducted in the absence of any commercial or financial relationships that could be construed as a potential conflict of interest.

## Footnotes

1 http://www.ensembl.org

2 http://www.ncbi.nlm.nih.gov/refseq

3 https://www.ncbi.nlm.nih.gov/CCDS

4 http://www.hgmd.cf.ac.uk/ac/index.php

5 http://hapmap.ncbi.nlm.nih.gov

6 http://www.1000genomes.org

7 http://evs.gs.washington.edu/EVS

8 http://gnomad.broadinstitute.org

9 http://exac.broadinstitute.org

10 http://www.hgvs.org

11 https://mutalyzer.nl

12 http://bg.upf.edu/fannsdb

13 www.mutationtaster.org

14 http://provean.jcvi.org/index.php

15 http://genetics.bwh.harvard.edu/pph2

16 http://www.uniprot.org

17 https://string-db.org

18 https://smart.embl.de

19 https://www.ncbi.nlm.nih.gov/projects/dbvar/clingen

## References

[B1] Abou Tayoun A. N. Pesaran T. DiStefano M. T. Oza A. Rehm H. L. Biesecker L. G. (2018). Recommendations for interpreting the loss of function PVS1 ACMG/AMP variant criterion. *Hum. Mutat.* 39 1517–1524. 10.1002/humu.23626 30192042 PMC6185798

[B2] Alcalde M. Campuzano O. Berne P. Garcia-Pavia P. Doltra A. Arbelo E. (2014). Stop-gain mutations in PKP2 are associated with a later age of onset of arrhythmogenic right ventricular cardiomyopathy. *PLoS One* 9:e100560. 10.1371/journal.pone.0100560 24967631 PMC4072667

[B3] Amendola L. M. Jarvik G. P. Leo M. C. McLaughlin H. M. Akkari Y. Amaral M. D. (2016). Performance of ACMG-AMP variant-interpretation guidelines among nine laboratories in the clinical sequencing exploratory research consortium. *Am. J. Hum. Genet.* 98 1067–1076. 10.1016/j.ajhg.2016.03.024 27181684 PMC4908185

[B4] Basso C. Burke M. Fornes P. Gallagher P. J. de Gouveia R. H. Sheppard M. (2008). Guidelines for autopsy investigation of sudden cardiac death. *Virchows Arch.* 452 11–18. 10.1007/s00428-007-0505-5 17952460

[B5] Brion M. Allegue C. Santori M. Gil R. Blanco-Verea A. Haas C. (2012). Sarcomeric gene mutations in sudden infant death syndrome (SIDS). *Forensic. Sci. Int.* 219 278–281. 10.1016/j.forsciint.2012.01.018 22361390

[B6] Brion M. A. C. Gil R. Torres M. Santori M. Poster S. Madea B. (2009). Involvement of hypertrophic cardiomyopathy genes in sudden infant death syndrome (SIDS). *Forensic. Sci. Int. Genet. Suppl. Ser.* 2 495–496.

[B7] Campuzano O. Allegue C. Fernandez A. Iglesias A. Brugada R. (2015a). Determining the pathogenicity of genetic variants associated with cardiac channelopathies. *Sci. Rep.* 5:7953. 10.1038/srep07953 25608792 PMC4302303

[B8] Campuzano O. Fernandez-Falgueras A. Sarquella-Brugada G. Sanchez O. Cesar S. Mademont I. (2015b). A genetically vulnerable myocardium may predispose to myocarditis. *J. Am. Coll. Cardiol.* 66 2913–2914. 10.1016/j.jacc.2015.10.049 26718681

[B9] Campuzano O. Allegue C. Partemi S. Iglesias A. Oliva A. Brugada R. (2014a). Negative autopsy and sudden cardiac death. *Int. J. Legal Med.* 128 599–606. 10.1007/s00414-014-0966-4 24532175

[B10] Campuzano O. Sarquella-Brugada G. Mademont-Soler I. Allegue C. Cesar S. Ferrer-Costa C. (2014b). Identification of genetic alterations, as causative genetic defects in long QT syndrome, using next generation sequencing technology. *PLoS One* 9:e114894. 10.1371/journal.pone.0114894 25494010 PMC4262446

[B11] De Bortoli M. Calore C. Lorenzon A. Calore M. Poloni G. Mazzotti E. (2017). Co-inheritance of mutations associated with arrhythmogenic cardiomyopathy and hypertrophic cardiomyopathy. *Eur. J. Hum. Genet.* 25 1165–1169. 10.1038/ejhg.2017.109 28699631 PMC5602010

[B12] Deo R. Albert C. M. (2012). Epidemiology and genetics of sudden cardiac death. *Circulation* 125 620–637. 10.1161/CIRCULATIONAHA.111.023838 22294707 PMC3399522

[B13] Fressart V. Duthoit G. Donal E. Probst V. Deharo J. C. Chevalier P. (2010). Desmosomal gene analysis in arrhythmogenic right ventricular dysplasia/cardiomyopathy: spectrum of mutations and clinical impact in practice. *Europace* 12 861–868. 10.1093/europace/euq104 20400443

[B14] Giudicessi J. R. Ackerman M. J. (2013). Determinants of incomplete penetrance and variable expressivity in heritable cardiac arrhythmia syndromes. *Transl. Res.* 161 1–14. 10.1016/j.trsl.2012.08.005 22995932 PMC3624763

[B15] Gopinathannair R. Etheridge S. P. Marchlinski F. E. Spinale F. G. Lakkireddy D. Olshansky B. (2015). Arrhythmia-Induced cardiomyopathies: mechanisms, recognition, and management. *J. Am. Coll. Cardiol.* 66 1714–1728. 10.1016/j.jacc.2015.08.038 26449143 PMC4733572

[B16] Hershberger R. E. Givertz M. M. Ho C. Y. Judge D. P. Kantor P. F. McBride K. L. (2018). Genetic evaluation of cardiomyopathy: a clinical practice resource of the american college of medical genetics and genomics (ACMG). *Genet. Med.* 20 899–909. 10.1038/s41436-018-0039-z 29904160

[B17] Kannankeril P. J. Bhuiyan Z. A. Darbar D. Mannens M. M. Wilde A. A. Roden D. M. (2006). Arrhythmogenic right ventricular cardiomyopathy due to a novel plakophilin 2 mutation: wide spectrum of disease in mutation carriers within a family. *Heart Rhythm* 3 939–944. 10.1016/j.hrthm.2006.04.028 16876743

[B18] Kapplinger J. D. Ackerman M. J. (2016). Founder mutation genotyping and sudden cardiac arrest: the promise of precision medicine fulfilled or the next step into precise uncertainty. *Circ. Cardiovasc. Genet.* 9 107–109. 10.1161/CIRCGENETICS.116.001387 27094198

[B19] Kobayashi Y. Yang S. Nykamp K. Garcia J. Lincoln S. E. Topper S. E. (2017). Pathogenic variant burden in the ExAC database: an empirical approach to evaluating population data for clinical variant interpretation. *Genome Med.* 9:13. 10.1186/s13073-017-0403-7 28166811 PMC5295186

[B20] Lek M. Karczewski K. J. Minikel E. V. Samocha K. E. Banks E. Fennell T. (2016). Analysis of protein-coding genetic variation in 60,706 humans. *Nature* 536 285–291. 10.1038/nature19057 27535533 PMC5018207

[B21] Marcus F. I. McKenna W. J. Sherrill D. Basso C. Bauce B. Bluemke D. A. (2010). Diagnosis of arrhythmogenic right ventricular cardiomyopathy/dysplasia: proposed modification of the task force criteria. *Circulation* 121 1533–1541. 10.1161/CIRCULATIONAHA.108.840827 20172911 PMC2860804

[B22] Marian A. J. Braunwald E. (2017). Hypertrophic Cardiomyopathy: genetics, pathogenesis, clinical manifestations, diagnosis, and therapy. *Circ. Res.* 121 749–770. 10.1161/CIRCRESAHA.117.311059 28912181 PMC5654557

[B23] Milano A. Blom M. T. Lodder E. M. van Hoeijen D. A. Barc J. Koopmann T. T. (2016). Sudden cardiac arrest and rare genetic variants in the community. *Circ. Cardiovasc. Genet.* 9 147–153. 10.1161/CIRCGENETICS.115.001263 26800703

[B24] Morini E. Sangiuolo F. Caporossi D. Novelli G. Amati F. (2015). Application of next generation sequencing for personalized medicine for sudden cardiac death. *Front. Genet.* 6:55 10.3389/fgene.2015.00055 PMC4345839 25784923

[B25] Nykamp K. Anderson M. Powers M. Garcia J. Herrera B. Ho Y. Y. (2017). Sherloc: a comprehensive refinement of the ACMG-AMP variant classification criteria. *Genet. Med.* 19 1105–1117. 10.1038/gim.2017.37 28492532 PMC5632818

[B26] Priori S. G. Blomstrom-Lundqvist C. (2015). 2015 European Society of cardiology guidelines for the management of patients with ventricular arrhythmias and the prevention of sudden cardiac death summarized by co-chairs. *Eur. Heart J.* 36 2757–2759. 10.1093/eurheartj/ehv445 26745817

[B27] Richards S. Aziz N. Bale S. Bick D. Das S. Gastier-Foster J. (2015). Standards and guidelines for the interpretation of sequence variants: a joint consensus recommendation of the american college of medical genetics and genomics and the association for molecular pathology. *Genet. Med.* 17 405–424. 10.1038/gim.2015.30 25741868 PMC4544753

[B28] Sarquella-Brugada G. Campuzano O. Cesar S. Iglesias A. Fernandez A. Brugada J. (2016). Sudden infant death syndrome caused by cardiac arrhythmias: only a matter of genes encoding ion channels? *Int. J. Legal Med.* 130 415–420. 10.1007/s00414-016-1330-7 26872470

[B29] Sinard J. H. (2013). Accounting for the professional work of pathologists performing autopsies. *Arch. Pathol. Lab. Med.* 137 228–232. 10.5858/arpa.2012-0012-CP 23368865

[B30] Straus S. M. Bleumink G. S. Dieleman J. P. van der Lei J. Stricker B. H. Sturkenboom M. C. (2004). The incidence of sudden cardiac death in the general population. *J. Clin. Epidemiol.* 57 98–102. 10.1016/S0895-4356(03)00210-5 15019016

[B31] Syrris P. Ward D. Asimaki A. Sen-Chowdhry S. Ebrahim H. Y. Evans A. (2006). Clinical expression of plakophilin-2 mutations in familial arrhythmogenic right ventricular cardiomyopathy. *Circulation* 113 356–364. 10.1161/CIRCULATIONAHA.105.561654 16415378

[B32] van der Zwaag P. A. Cox M. G. van der Werf C. Wiesfeld A. C. Jongbloed J. D. Dooijes D. (2010). Recurrent and founder mutations in the Netherlands : plakophilin-2 p.Arg79X mutation causing arrhythmogenic right ventricular cardiomyopathy/dysplasia. *Neth. Heart J.* 18 583–591. 10.1007/s12471-010-0839-5 21301620 PMC3018603

[B33] Wang J. Wan K. Sun J. Li W. Liu H. Han Y. (2018). Phenotypic diversity identified by cardiac magnetic resonance in a large hypertrophic cardiomyopathy family with a single MYH7 mutation. *Sci. Rep.* 8:973. 10.1038/s41598-018-19372-4 29343710 PMC5772531

